# Supplementary material for: Radiographers’ perceptions on the quality of managing general radiographic paediatric examinations through the use of a reflective tool
Source: PLoS One. 2023 Dec 7;18(12):e0295603. doi: 10.1371/journal.pone.0295603 (PMC10703265; doi:10.1371/journal.pone.0295603)
Supplement: S1 Checklist — (PDF) [file pone.0295603.s001.pdf]

A quality management checklist for paediatric imaging examinations

This is to be completed by the radiographer in relation to a general overview of the departments processes for general paediatric radiographic examinations. From your experience of conducting general paediatric radiographic examinations, undertaking this questionnaire will allow you to reflect on the entire procedure to help improve the quality of the imaging as well as the services delivered. Answer with a ‘yes’ or ‘no’ and provide information in the ‘Recommended action plan’ on ways each section could be improved for general paediatric radiographic examinations. Additional comments can be made in the ‘Other comments’ section for each item.

Elements	Descriptors	Yes	No	Recommended action plan	Other comments
**Ease of access to services and the service delivery**	Resources for information (e.g. website to inform patient decisions on practice or examinations; patients access to education programmes and information leaflets).				
	Appointment scheduling and emergency / urgent access to imaging.				
	Navigation from home to point of face-to-face contact to the radiology department (e.g. parking, signage, web information).				
	Documentation and verification / record keeping (request orders are appropriately completed and filed, access to old imaging examinations and reports).<br>a. Within the radiology department.				

Elements	Descriptors	Yes	No	Recommended action plan	Other comments
	b. Other healthcare professionals (e.g. means to obtain and share previous and current imaging with each part of the patient healthcare team such as nurses, referring doctor, etc.).				
**Efficiency of task performance**	<u>Pre-examination</u><br>a. Appropriateness and quality of request.				
	b. Patient identification verified.				
	c. Explained examination to patient / parent.				
	d. Verified pregnancy / last menstrual period (LMP), if appropriate.				
	<u>Examination</u><br>a. Beam body part image receptor aligned (with or without grid use).				
	b. SID checked.				

Elements	Descriptors	Yes	No	Recommended action plan	Other comments
	c. Patient body part positioned and collimation applied.				
	d. Anatomical marker placement.				
	e. Shielding, where applicable.				
	f. Appropriate application of immobilisation aid, where applicable (e.g. physical, chemical, mechanical, psychological).				
	g. Exposure technique selection.				
	h. Final adjustment if necessary (e.g. rotation, tilt, etc.).				
	i. Exposure taken.				
	<u>Post examination</u><br>a. Processing and image review (e.g. acceptable range of image quality, artifacts).				
	b. Decision to repeat or proceed to archiving and ready for reporting.				

Elements	Descriptors	Yes	No	Recommended action plan	Other comments
**Patient care and wellbeing (include family and caregiver)**	Care communication and interactions (issues of literacy, language proficiency, physical interaction, touching / visual stimuli, how the child communicates).				
	Child's interests (e.g. to assess what works and could calm the child, check reaction to sound, light).				
	Assessing cognitive impairment and stage of development (Sensitometer / preoperational stage / concrete operational stage / formal operational stage).				
	Socio-cultural and emotional considerations.				
	Power relations between the patient, accompanying adults and radiographer (e.g. radiographer is in a more powerful position than the patient).				
	Space / opportunity for decisions, discussions, consent, information exchange with patient to allow an understanding of the patient's needs.				
	Behaviour of staff in an appropriate demeanour.				

Elements	Descriptors	Yes	No	Recommended action plan	Other comments
**Ethical consideration**	Child's rights / risks versus harm / benefits.				
	Ethical and legal guidelines for the care of adolescents regarding privacy and medical decision-making.				
	Consent consideration and assent based on the child's age and developmental understanding.				
	Guidelines for appropriate age and sex of the patient, especially considering adults who may be accompanying minors.				
**Staff related elements**	Paediatric medical imaging equipment and accessories is compliant (e.g. equipment size, space and appropriate function status).				
	Mechanism of reporting errors (e.g. incident writing).				
	Patient data utilisation (e.g. reject-film analysis, ordering of equipment).				

Elements	Descriptors	Yes	No	Recommended action plan	Other comments
	Evaluation of patient, family and or caregiver satisfaction (e.g. professionalism, pacing the examination, efficiency of task performance, equitable treatment).				
	Education programmes for patients, students and staff.				
	Peer debriefing.				
	Professional development of staff.				
	Multidisciplinary team engagement and regular review throughout the institution for paediatric medical imaging (e.g. including occupational health and safety).				
**Quality management**	Safety (cultural, infection, radiation safety, contrast agents, communication, safe patient environment, incident reporting which could include patient handling as well as radiation dose incidents).				

Elements	Descriptors	Yes	No	Recommended action plan	Other comments
	Stakeholder participation in relation to quality improvement measures (internal stakeholders that operate within the institution such as management team, employees, patients and external stakeholders that operate outside the institution such as external funders, professional bodies, accrediting bodies).				
	Institutional culture on safety management (e.g. error disclosure and risk management).				
	Monitoring of quality improvement (e.g. through quality assurance, quality control of equipment and workplace with paediatric focus).				
	Standards and compliance with authorising institutional and national regulatory authorities.				
	Paediatric medical imaging procedure protocols, policies and guidelines.<br>a. According to developmental needs (e.g. Skeletal survey accidental and non-accidental injuries).				
	b. Dose reference levels.				

Elements	Descriptors	Yes	No	Recommended action plan	Other comments
	c. Unique needs of paediatric patients.				
	d. Timely advanced level of care.				
	e. Internal and external institutional referral for efficient safe transitioning.				
	Involving families and the community in quality assurance, policy development and facility improvements.				
