# Supplementary material for: Radiographers’ perceptions on the quality of managing general radiographic paediatric examinations through the use of a reflective tool
Source: PLoS One. 2023 Dec 7;18(12):e0295603. doi: 10.1371/journal.pone.0295603 (PMC10703265; doi:10.1371/journal.pone.0295603)
Supplement: Attachment — Submitted filename: Response to Reviewers.docx [file pone.0295603.s002.docx]

**Rebuttal Letter**

**Original Manuscript ID:** PONE-D-23-26933

**Original Article Title:** “Radiographers’ perceptions on the quality of managing general radiographic paediatric examinations through the use of a reflective tool”

**To:** PLOS ONE

**Re:** Response to Reviewers

Dear Editor,

Thank you for allowing a resubmission of our manuscript, with an opportunity to address the reviewers’ comments.

We are uploading

(1) Our point-to-point response to the comments below (Response to Reviewers)

(2) A marked-up copy with track changes (Revised Manuscript with Track Changes)

(3) An unmarked version without track changes (Manuscript)

Kind regards,

Kate Caruana

Corresponding Author

Medical Radiation Practitioner

Email: [kate.caruana@act.gov.au](mailto:kate.caruana@act.gov.au)

Tel No: (+61) 498 108 772

Date: 14 November 2023

**Reviewer#1, Concern #1:**

**Reviewer’s comment:** The authors should try to highlight the gap in literature that the study sought to address in the introduction and they should also discuss the implications for practice in the discussion.

**Authors’ response:** We have revised the introduction and discussion, adding some information regarding the gap of literature and implications for practice.

**Reviewer#1, Concern #2:**

**Reviewer’s comment:** The authors discuss the reflective tool in the Materials and techniques section. Even while reflection is addressed, it is unclear where the qualitative technique fits in. The author should be more precise in defining and specifying the inclusion of the qualitative component in their study by showing that 'open-ended questions.' were used in section two.

**Authors’ response:** We have addressed this concern in the materials and methods section, clearly showing that open-ended questions were used in section two of the questionnaire.

**Reviewer#1, Concern #3:**

**Reviewer’s comment:** The response rate was quite low. The author should include brief details for the reasons.

**Authors’ response:** This has been previously addressed in the discussion within the limitations of the study. We have revised this section.

**Reviewer#1, Concern #4:**

**Reviewer’s comment:** The conclusion of the study could be improved to summarise the highlights of the study better. The current conclusion does not summarise the findings well. The authors could indicate non-generalisability of the findings more so, because of the response rate. Also, indicate areas for future research and the importance of using the tool.

**Authors’ response:** Many thanks for your kind suggestion. We have rigorously revised the conclusion, summarising the key findings as well as providing information regarding future research.

NOTE: We have further addressed the Journal Requirements as follows:

**Concern #1:**

1. Please ensure that your manuscript meets PLOS ONE's style requirements, including those for file naming. The PLOS ONE style templates can be found at

<https://journals.plos.org/plosone/s/file?id=wjVg/PLOSOne_formatting_sample_main_body.pdf> and

<https://journals.plos.org/plosone/s/file?id=ba62/PLOSOne_formatting_sample_title_authors_affiliations.pdf>

**Authors’ response:** We have reviewed and followed the PLOS ONE’S style and format in the revised manuscript.

**Concern #2:**

2. We note that you have indicated that data from this study are available upon request. PLOS only allows data to be available upon request if there are legal or ethical restrictions on sharing data publicly. For information on unacceptable data access restrictions, please see http://journals.plos.org/plosone/s/data-availability#loc-unacceptable-data-access-restrictions.

In your revised cover letter, please address the following prompts:

a) If there are ethical or legal restrictions on sharing a de-identified data set, please explain them in detail (e.g., data contain potentially identifying or sensitive patient information) and who has imposed them (e.g., an ethics committee). Please also provide contact information for a data access committee, ethics committee, or other institutional body to which data requests may be sent.

b) If there are no restrictions, please upload the minimal anonymized data set necessary to replicate your study findings as either Supporting Information files or to a stable, public repository and provide us with the relevant URLs, DOIs, or accession numbers. Please see http://www.bmj.com/content/340/bmj.c181.long for guidelines on how to de-identify and prepare clinical data for publication. For a list of acceptable repositories, please see <http://journals.plos.org/plosone/s/data-availability#loc-recommended-repositories>.

**Authors’ response:** We have amended the cover letter to address the ethical/legal restrictions of sharing the data set.

**Concern #3:**

3. Please review your reference list to ensure that it is complete and correct. If you have cited papers that have been retracted, please include the rationale for doing so in the manuscript text, or remove these references and replace them with relevant current references. Any changes to the reference list should be mentioned in the rebuttal letter that accompanies your revised manuscript. If you need to cite a retracted article, indicate the article’s retracted status in the References list and also include a citation and full reference for the retraction notice.

**Authors’ response:** We have reviewed the reference list and ensured its correctness and completeness. We have added and amended the reference list with additional papers to enhance the literature gap as requested by the reviewer. Whilst no papers were removed/retracted from the reference list as all papers were available, one reference was updated to reflect the latest best practice.
